# Ethics and biodiversity offsetting

**DOI:** 10.1111/cobi.13603

**Published:** 2020-10-08

**Authors:** Mikael Karlsson, Karin Edvardsson Björnberg

**Affiliations:** ^1^ Division of Philosophy KTH Royal Institute of Technology Teknikringen 76 Stockholm SE‐100 44 Sweden

**Keywords:** anthropocentrism, commodification of nature, ecological compensation, instrumental value, intrinsic value, nature conservation, nonanthropocentrism, social justice, antropocentrismo, compensación ecológica, conservación de la naturaleza, justicia social, mercantilización de la naturaleza, no antropocentrismo, valor instrumental, valor intrínseco, 人类中心主义, 非人类中心主义, 自然的商品化, 生态补偿, 工具价值, 内在价值, 自然保护, 社会公正

## Abstract

Biodiversity offsetting is an increasingly applied tool aiming to compensate for environmental damage caused by exploitation projects. Critics, however, raise concerns over the purported effectiveness of offsetting and question the ethical underpinnings and implications of offsetting. These ethical dimensions have largely been overlooked in research, which may lead to offsetting systems that fail to respect the values intended to be safeguarded. To address these dimensions, 5 ethical objections in the scientific literature were identified: offsetting violates nature's intrinsic value; losses of nature cannot be compensated for by human interventions; too little is known to make adequate trades; offsetting impedes virtuous dispositions toward nature; and offsetting has negative justice implications. We examined these objections and arguments against them based on the ethical concepts of intrinsic and instrumental values, anthropocentrism, nonanthropocentrism, and deontological, consequentialist, and virtue‐ethical paradigms. Both nonanthropocentric and anthropocentric concerns were expressed in deontological, consequential, and virtue‐ethical framings. Objections mostly had a deontological or virtue‐ethical basis, whereas counterarguments were based on consequential reasoning, but common ground in practice is often conceivable. Based on our findings, we formulated 10 recommendations for policy makers and 5 questions for practitioners to consider. We propose, for example, that policy makers clarify aims, legislate on no‐go areas, and govern the use of multipliers. We suggest that practitioners consider, for instance, how to improve case‐specific knowledge and promote learning and stakeholder engagement. We hope these recommendations and questions will encourage further discussion of the ethics of biodiversity offsets and ultimately strengthen the respect for biodiversity and human‐welfare values at stake in offsetting projects.

## Abstract 2

Article impact statement: Five ethical objections should be considered in the design and implementation of biodiversity offsetting.

## Abstract (es)

Ética y Compensación de Biodiversidad

**Resumen**

La compensación de la biodiversidad es una herramienta que cada vez se aplica más a la búsqueda de la remuneración por el daño ambiental causado por proyectos de explotación. Sin embargo, hay quienes la critican basándose en la presunta efectividad de las compensaciones y cuestionan los sustentos éticos y las implicaciones de las compensaciones. Estas dimensiones éticas han sido ignoradas en la investigación, lo cual puede resultar en sistemas que no respetan los valores que se pretende salvaguardar. Para resolver estas dimensiones identificamos cinco objeciones éticas en la literatura: las compensaciones violan el valor intrínseco de la naturaleza; las pérdidas de la naturaleza no pueden compensarse mediante intervenciones humanas; se conoce muy poco para realizar intercambios adecuados; las compensaciones impiden las disposiciones virtuosas hacia la naturaleza; y las compensaciones tienen implicaciones de justicia negativa. Analizamos estas objeciones y los argumentos en su contra basados en los conceptos éticos de los valores intrínsecos e instrumentales, el antropocentrismo, el no antropocentrismo y los paradigmas deontológico, consecuencialista y de virtud ética. Tanto las preocupaciones antropocéntricas como las no antropocéntricas se expresaron mediante encuadres deontológicos, consecuencialistas y de virtud ética. Las objeciones principalmente tuvieron una base deontológica o de virtud ética, mientras que los contraargumentos estuvieron basados en el razonamiento consecuencial pero los puntos de coincidencia en la práctica con frecuencia son concebibles. Con base en nuestros hallazgos formulamos diez recomendaciones para los formuladores de políticas y cinco preguntas para los practicantes de la conservación para que las tomen a consideración. Por ejemplo, proponemos que los formuladores de políticas clarifiquen los objetivos, legislen las áreas prohibidas y determinen el uso de multiplicadores. Sugerimos que los practicantes consideren cómo mejorar el conocimiento específico por caso y promover el aprendizaje y la participación de los actores sociales. Esperamos que estas recomendaciones y preguntas fomenten una mayor discusión sobre la ética de las compensaciones de biodiversidad y, finalmente, fortalezcan el respeto por la biodiversidad y los valores de bienestar humano en juego en los proyectos de compensaciones.

## 摘要

生物多样性补偿旨在补偿开发项目造成的环境破坏, 正日益得到应用。然而, 有批评者对补偿的有效性提出了担忧, 并对其道德基础和作用提出了质疑。以往研究很大程度忽视了这些伦理维度, 这可能导致生物多样性补偿系统没有尊重其旨在维护的价值。为了解决这些问题, 我们通过科学文献综述确定了五项伦理上的反对意见: 补偿机制违背了自然的内在价值; 人类干预不能补偿自然的损失; 人们所知甚少, 无法进行适当的交易补偿; 补偿损害了对自然的善意; 补偿会带来负面的影响。我们基于内在价值和工具价值的伦理概念、人类中心主义、非人类中心主义、道义论、结果论和美德 ‐ 伦理范式, 检验了这些反对意见和对其的争论。非人类中心主义和人类中心主义的关注点都在道义论、结果论和美德 ‐ 伦理框架中得到了表达。反对意见大多有道义论或美德 ‐ 伦理基础, 而对它们的回应则基于结果论推理, 但通常在实践中双方存在可能的共同基础。基于我们的研究发现, 我们向政策制定者提出了 10 条建议, 并对实践者提出了 5 个问题供其考虑。例如, 我们建议决策者明确目标, 立法确定禁区, 管理乘数的使用。我们还建议实践者思考如何积累具体案例的知识、促进学习和推动利益相关者参与。我们希望这些建议和问题将鼓励人们进一步讨论生物多样性补偿的伦理问题, 并最终加强在补偿项目中对生物多样性和人类福利价值的尊重。**【翻译: 胡怡思; 审校: 聂永刚】**

## Introduction

Biodiversity offsetting is a nature‐conservation policy tool aimed at counterbalancing the adverse environmental impacts of exploitation by generating at least equivalent compensatory conservation benefits. The oft‐stated goal is to achieve no net loss and preferably a net gain of biodiversity (BBOP 2012a). The tool originates from U.S. pollution regulations (Bonneuil 2015; Coralie et al. 2015), from which it spread to other management fields, such as forest and wildlife conservation. At present, over 60 (Maron et al. 2016), and perhaps over 100 (Bull & Strange 2018), countries have offsetting policies in place, including the United States, Canada, Australia, China, and several European Union member states (Bennett & Gallant 2017). Although emphasized as a valuable conservation tool by many (e.g., Reid 2013; Santos et al. 2015; Levrel et al. 2017), biodiversity offsetting attracts criticism. Much of the criticism targets ecological, technical, or governance aspects of policy, such as substandard performance of some established offsetting practices (Kujala et al. 2015; Lindenmayer et al. 2017; May et al. 2017); difficulties involved in quantifying and comparing different ecological units (Walker et al. 2009; Reid 2013; Goncalves et al. 2015); and challenges associated with monitoring and evaluating offsetting schemes over time (Reid 2013; Santos et al. 2015; Schoukens & Cliquet 2016). However, the ethical issues involved, often underlying much of the criticism, have received much less attention. One important exception is an article by Ives and Bekessy (2015), who explore the linkages between ethical theories and, not least, how to measure biodiversity and how to determine its value. The limited attention generally given to the ethics of biodiversity offsetting is unfortunate because ethical analysis is needed for developing and implementing policies that truly safeguard the values at stake. We aimed to address this shortcoming by identifying and analyzing common ethical objections to biodiversity offsetting mentioned in the scientific literature. By formulating recommendations for policy makers and questions for practitioners to consider, we also aimed to encourage more attention to the ethical underpinning of the values potentially affected by biodiversity offsets. In particular, we investigated biodiversity offsetting with respect to the following questions: What types of values and objects are considered to be at stake? Which ethical paradigms may underlie the normative concerns that are raised? What are the main objections and counterarguments in the scientific literature? And, what should policy makers and practitioner consider in this context?

Because we considered both researchers and nonresearchers, including those with a background in philosophy as well as those with a background in biology, our presentation is nontechnical and interdisciplinary. The recommendations and questions we formulated should be relevant for a broad group of stakeholders and should facilitate decision making that is ethically well grounded. Our analysis of ethical objections to biodiversity offsetting is broader than in previous scientific studies and is based on fundamental ethical concepts and paradigms. Further, the recommendations for policy makers and the questions for practitioners are, to our knowledge, not formulated in a structured manner elsewhere in the scientific literature.

### Literature Search and Study Selection

The core of our study consisted of an analysis of ethical objections to biodiversity offsetting found in scientific articles identified in Scopus using the following summarizing search string: ((TITLE‐ABS‐KEY (“biodiversity offset*”) OR TITLE‐ABS‐KEY (“*ecological compensation*”)) AND (TITLE‐ABS‐KEY (ethic* OR moral* OR value*) OR TITLE‐ABS‐KEY (*justice* OR distribut*) OR TITLE‐ABS‐KEY (*social* AND (*impact* OR *effect* OR consequence*)))) OR (TITLE‐ABS‐KEY (“*ecosystem services*”) AND TITLE‐ABS‐KEY (ethic* OR moral* OR ((*intrinsic* OR *instrumental*) AND value*))).

The search string generated 874 document hits (up to 6 November 2019). To identify a set of articles of high relevance and manageable size, a selection in 2 steps was done. First, the list of hits was filtered based on studying document titles. Publications not written in English or not focusing on offsetting or ethical issues were excluded. Both M.K. and K.E.B. rated each of the remaining 138 articles independently as of high, medium, or low relevance based on reading document abstracts. Publications that at least one author considered of high relevance, but that neither author deemed of low relevance were selected. This resulted in 37 articles, which were analyzed by applying the ethical concepts and paradigms described in “Ethical Analyses.” Some other scientific publications and gray literature were also included, for example, on the history of offsetting.

### Ethical Analyses

Certain ethical concepts were employed in a few previous studies of biodiversity offsetting (Ives & Bekessy 2015; Maron et al. 2016), but by applying a multidimensional set of ethical concepts and paradigms, the arguments can be further characterized. Conceptual distinctions help describe the values and objects at stake, as well as the underlying normative paradigms. We used the concepts of instrumental and intrinsic values, anthropocentrism and nonanthropocentrism, and deontological, consequentialist, and virtue‐ethical paradigms. Instrumental value and intrinsic value express different ideas about what kind of value things like nonhuman organisms, species, habitat, and geophysical processes can have (Hargrove 1989). Entities considered valuable as means to other ends are referred to as instrumentally valuable because they contribute to achieving something else that is considered valuable. Entities valuable in themselves, for their own sake, are called intrinsically valuable. An entity can have both instrumental and intrinsic value. A wetland, for example, can be instrumentally valuable if it mitigates nitrogen leakage, but it can also be considered valuable in itself.

Anthropocentrism and nonanthropocentrism represent different ideas about what entities have intrinsic value and thus can be considered ethically relevant. Anthropocentrism means that only humans and human fulfillment, understood as happiness, welfare, or having a good life in some sense, are considered valuable in themselves. Nonanthropocentrism extends such value, at least to some degree, to nonhuman organisms or other biological entities, such as species. It does not, per se, rule out the idea that humans have intrinsic value.

Deontological, consequentialist, and virtue ethical paradigms concern what is the right or wrong thing to do. Hansson (2007) summarizes these 3 models of reasoning with 3 metaphors: a fence, a scale, and a compass. When several courses of action are available, deontological ethics set certain absolute limits for what is morally permissible to do, which should never be exceeded, regardless of the consequences. Consequentialist (or, more particularly, utilitarian) ethics weigh the advantages and disadvantages of each course of action and choose the alternative with the greatest net advantage. Classical utilitarianism requires that one maximize expected utility, or the good, conceived as happiness. Virtue ethics is less occupied with what is right or wrong but focuses on the actor's character or proclivity to act in certain ways. Anthropocentric and nonanthropocentric arguments for protecting ethically relevant objects may make use of any of these ethical paradigms.

We applied the described ethical concepts and paradigms in the analysis of the selected articles.

## Five Ethical Objections to Biodiversity Offsetting

### Offsetting Violates Nature's Intrinsic Value

The first main objection identified in the selected literature is that biodiversity offsetting itself is wrong, regardless of what positive or negative consequences it may bring about. Commodifying nature (i.e., dividing it into units that are bought, sold, or otherwise traded) treats it as a means to human ends and not as an end in itself (Spash 2015; Spash & Aslaksen 2015; Millward‐Hopkins 2016). This violates nature's intrinsic value. According to this view, nature should be protected for its own sake, not merely based on how much welfare it can generate. This is a nonanthropocentric deontological argument. For example, the UN (1982) World Charter for Nature states that “[e]very form of life is unique, warranting respect regardless of its worth to man, and, to accord other organisms such recognition, man must be guided by a moral code of action.”

However, the idea that offsetting through commodification of nature violates its intrinsic value has been challenged in the literature. Caney (2010) argues that, although nature may have intrinsic value and may be priceless in a philosophical sense, commodification through monetization may still be the best means of safeguarding this value in the real world. Despite implementation challenges, offsetting and trading are powerful tools, according to Santos et al. (2015). This consequential ethical approach may be either anthropocentric or nonanthropocentric, but often translates the intrinsic value of nature into “the language of economics” (McCauley 2006), implying a utilitarian anthropocentric ethic. Some, however, suggest that offsetting cannot be seen as “marketization” of nature (Vaissière et al. 2017), and the associated risks have, in empirical studies, been judged to be more “theoretical than… real” (Levrel et al. 2017). To prevent the overriding of the putative intrinsic values in nature, proponents advocate for the so‐called mitigation hierarchy (i.e., the sequential steps to follow before turning to offsetting as a last resort): avoidance, minimization, and rehabilitation or restoration (McKenney & Kiesecker 2010).

### Losses of Nature Cannot be Compensated for by Human Interventions

Biodiversity offsetting is wrong because it is based on an allegedly false ontological assumption that loss of natural value can be compensated for by creating at least equally valuable units of nature. Different versions of the objection exist in the literature.

At the most stringent end of the spectrum are those who question whether true fungibility exists at all for biodiversity components (Salzman & Ruhl 2000; Moreno‐Mateos et al. 2015). Humans cannot restore life to an individual bird, de‐extinct species (so far), or replant an old‐growth forest to duplicate its original state. Because no 2 organisms or places will ever be identical, such losses can never truly be offset (Maron et al. 2016). Believing that offsetting creates win‐win solutions to the problem of biodiversity loss is, therefore, a distortion of reality. This deontological nonanthropocentric view leads to peremptorily rejecting offsetting altogether. However, fungibility in this strict sense is not what offsetting always aims for. The purpose is not necessarily to trade off one unit of nature for its twin elsewhere. On the contrary, offsetting is typically based on the understanding that biodiversity components are sometimes inevitably lost due to exploitation. Offsets then help to make up for the lost values as far as possible (Morris et al. 2006), which fits well within a consequential nonanthropocentric or anthropocentric paradigm.

A less absolutist and more common way of framing the objection is to claim that many losses of nature are, if not impossible, then at least very difficult to offset, even though it sometimes may be possible in particular in‐kind and in‐situ circumstances. For attributes, such as pristineness (i.e., nature untouched by humans), it follows by definition that losses cannot be offset other than through purchasing another pristine unit of nature (Cowell 2000). The argument that there are limits to what can be compensated for rests on a deontological nonanthropocentric basis. It is often used to advocate restrictions on how trades can be made, even though advocates may concede that some natural values can be fostered by humans, for example, by setting aside areas otherwise targeted for exploitation. An opposing view, defended on consequentialist no‐anthropocentric grounds, is that compensation can be achieved also out‐of‐kind or ex situ. To what extent such flexibility should be allowed is debated. The International Business and Biodiversity Offsets Program (BBOP 2012a) recommends that offsets be in‐kind; that is, “gains from the biodiversity offset are for the same or very similar biodiversity components to those impacted” (Bull et al. 2015). Presumably, this view rests on the assumption that value losses can be minimized if damages and offsets are as similar as possible. However, it is also claimed that conservation objectives are not always best served through in‐kind exchanges; instead, larger net gains may be created by allowing for greater flexibility (Habib et al. 2013).

A distinction is often made between 3 categories of flexibility: type, space, and time. Bull et al. (2015) caution against a generous application of flexibility of type because that could lead to some habitat types losing out if trade‐offs are not adequately coordinated across a landscape. Regarding space, Bull et al. (2015) acknowledge that flexibility “can lead to more efficient implementation of offset activities across a landscape”; however, it could also create social equity problems. The authors, moreover, regard the time parameter as important, considering how long it often takes to accrue ecological values starting from scratch, and they underline the risk for undesirably long time lags. To avoid losses associated with flexible trading schemes, so‐called “no go areas” could be defined in terms of assets that are strictly protected from encroachment (Walker et al. 2009) and thus exist within a deontological frame of restrictions. New South Wales BioBanking, for instance, applies the criteria of irreplaceability and vulnerability to identify “red flag areas,” where conservation values are high and impacts should be avoided (McKenney & Kiesecker 2010). As determinants of red‐flag areas, they mention “vegetation types and the estimated distribution remaining in the catchment management authority, the presence of critically endangered or endangered ecological communities, and the presence of threatened species.”

### Too Little is Known to Make Adequate Trades

It is wrong to allow biodiversity offsets when human knowledge of nature is so lacking. This objection shifts the focus onto epistemic issues. Even if one assumes that some losses of natural value can in principle be offset through human intervention, it may be argued that nature is so complex that it would be very difficult, or even impossible, for humans to identify and compensate for all values at stake (Ives & Bekessy 2015; Thebaud et al. 2015). Although it might be possible to recreate nature in a “narrow suite of habitats and species” (e.g., ruderal areas), older habitats are more difficult to offset (Morris et al. 2006). This may be particularly problematic in a longer time perspective if societal values or preferences change. Here, a difference is visible between this epistemically based argument, that compensation may be wrong due to lack of knowledge, and the ontologically based view, that certain things in nature are irreplaceable per se. Both views, however, are based on deontological nonanthropocentrism.

Some offsetting proponents raise the counterargument that epistemic challenges can be coped with by using so‐called multipliers (i.e., factors that increase an offset area to ensure no net loss or, better still, net gains) (BBOP 2012a). The basis for this approach is consequential ethical reasoning, whether anthropocentric or nonanthropocentric. Applying multipliers raises the demands on offsetting agents and increases the likelihood that various losses of natural value are adequately offset, even in situations characterized by epistemic uncertainty. Multipliers thus function as a kind of precautionary measure. However, in a review of multipliers in both theoretical literature and practical implementation, Bull et al. (2017a) reveal the existence of evident gaps in both theory and practice and propose applying much larger multipliers for promoting adequate trades, which may be acceptable at least when values are “secular” and not “sacred.” Problems of measuring and valuing biodiversity also challenge the use of multipliers and consequential reasoning, according to Ives and Bekessy (2015).

### Offsetting Impedes Virtuous Dispositions Toward Nature

Offsetting corrupts the character of moral agents. Thinking of the environment as a commodity could change human dispositions toward nature in an ethically problematic way. It risks creating the impression that there is nothing wrong with harming nature, as long as the harm is compensated (Reid 2013; Ives & Bekessy 2015; Rode et al. 2015; Maron et al. 2016). At the porous boundary between deontological and virtue ethics, such an attitude could make people believe that nature is worth protecting only when it is instrumentally valuable. Expansion of markets to the area of conservation policy can affect the ethical basis on which decisions are made, thereby crowding out deontological nonanthropocentric motivations (McCauley 2006; Millward‐Hopkins 2016). Moreover, from a virtue‐ethics perspective, commodification could prevent people from developing virtuous, caring, nonanthropocentric dispositions toward nature, seen as constitutive of a meaningful life. There is some empirical evidence supporting the argument that economic incentives can influence people's environmental attitudes and how people are disposed toward the environment, according to a review by Rode et al. (2015), even though more studies are called for.

Whether offsetting always engenders a predominantly corrupting impact on dispositions and motivations has, however, been questioned. Being forced to take part in offsetting could foster environmental concern, as Persson (2011) notes. Before commencing a project, a developer has to consider various means of avoiding, mitigating, and, if it comes to that, ultimately offsetting damage. Such reflection might strengthen the environmental sensitivity of developers in the planning process. However, Persson (2011) also notes that there are offsetting setups in which developers do not perform compensatory work (e.g., in habitat banking, when commercial intermediaries sell offsets to developers), making it less plausible that caring habits of action might develop.

### Offsetting has Negative Justice Implications

The distribution of offsetting costs and benefits can be unjust. The direct and indirect welfare implications may create winners and losers geographically and temporally and risk exacerbating inequities (Salzman & Ruhl 2000; Mandle et al. 2015; Scholte et al. 2016). Empirical studies on spatial equity implications of wetland mitigation banking show that benefits such as flood protection are often locally valuable, meaning that ex situ offsetting may be ethically problematic (BenDor et al. 2008; BenDor & Stewart 2011; Maron et al. 2016). Ruhl and Salzman (2006) worry about the tendency to move wetlands from urban to rural areas, where land is cheaper, so that “completely different populations are winners and losers.” To compensate for this, they propose granting enhanced credit allotments to banks that locate closer to the urban areas that experience losses. Another example concerns exploitation on the fringes of urban settlements that generate impaired access to green spaces by communities (Ives & Bekessy 2015; Sullivan & Hannis 2015). The spatial equity implications of offsetting schemes are relevant especially in the Global South. For instance, a study on the social impacts of 2 types of biodiversity offsets in Madagascar shows inequitable impacts at national, local, and household levels (Bidaud et al. 2017), with primarily rural communities on the edges of a rainforest bearing the costs of a mining project linked to forest conservation. A study of a proposed road project in the Peruvian Amazon indicates that, unless taken into account in the construction and mitigation processes, spatial inequities, in this case pertaining to drinking water quality, would be exacerbated (Mandle et al. 2015).

Spatial equity concerns again raise the issue of flexibilities. Apostolopoulou and Adams (2017) note that ex situ offsetting may be problematic because it is based on the assumption that “exchanges of ecological losses and gains can be separated from their ecological, cultural, socio‐economic, and political context” (Sullivan & Hannis 2015; Rogers & Burton 2017). By reframing nonhuman nature in terms of biodiversity units traded across space, their specific relation to place may be reworked. That could be considered unethical from an anthropocentric viewpoint, whether on deontological or consequential grounds. In the latter case, however, applying large enough multipliers could defend such offsetting. In line with that, a study comparing offsetting schemes in the United States, European Union, Australia, and Brazil shows that policy guidance is “trending away from strict requirements for in‐kind offsets located as close to impact sites as possible, in favor of identifying the most environmentally preferable offset options within the watershed or landscape” (McKenney & Kiesecker 2010).

Temporal redistributions in offsetting may also be seen as socially unjust (BenDor et al. 2008; Sullivan & Hannis 2015; Maron et al. 2016; Bidaud et al. 2017). In a study in New South Wales, Gibbons et al. (2018) estimate that a no‐net‐loss of native vegetation would not occur for 146 years. Such time lags between impacts and their offsets may result in intergenerational injustice because those who lose out in the near future will not be those who eventually benefit from the offset. To cope with such issues, the New Zealand Government's (2014) guidance on good practice acknowledges that temporal lags between loss and gain could necessitate using multipliers. However, selecting an appropriate multiplying factor is tricky because it is difficult to determine future‐compensation acceptance standards (Bull et al. 2017a). A way forward is proposed in the BBOP Standard, Principle 6: “In areas affected by the development project and by the biodiversity offset, the effective participation of stakeholders should be ensured in decision‐making about biodiversity offsets, including their evaluation, selection, design, implementation, and monitoring” (BBOP 2012b). This is particularly important because stakeholders may take views on offsetting principles that differ widely from what experts consider to be good practices (Bull et al. 2017b).

## Discussion

The literature we studied shows that offsetting is objected to because it is considered to violate nature's intrinsic value and because it is seen to harm humans, especially those who are poor, vulnerable, or excluded from decision making. Nonanthropocentric and anthropocentric concerns are variously expressed in deontological, consequential, and virtue‐ethical framings. A summary of the ethical characteristics of the 5 objections to biodiversity offsetting is given in Table 1.

**Table 1:** Ethical characteristics of objections to biodiversity offsetting

Objections	Deontological ethics	Consequential ethics	Virtue ethics	Nonanthropocentric values	Anthropocentric values
Offsetting violates nature's intrinsic value	X		X	X	
Losses of nature cannot be compensated for by human interventions	X			X	X
Too little is known to make adequate trades	X	X	X	X	X
Offsetting impedes virtuous dispositions toward nature			X	X	X
Offsetting has negative justice implications	X	X	X		X

The literature, moreover, shows a dividing line between those who more or less oppose offsetting, often on a deontological or virtue‐ethical basis, and those who raise counterarguments and argue for applying offsetting, mostly based on consequential reasoning. The main deontological objection is that offsets assume that nature is a mere means to human welfare and that offsets commodify nature. There are different versions of the commodification objection, but at its core lies the view that certain biodiversity components should not be up for trade (Edvardsson Björnberg 2020). There is, therefore, a need for exchange limits. From a virtue‐ethical standpoint, playing the pragmatic game implies that one loses the opportunity to relate to the world in other ways than through considerations of efficiency (Reid 2013). Although many who support a consequential ethical approach may acknowledge intrinsic values in nature, they may also consider offsetting an effective, perhaps necessary, conservation tool (BBOP 2012a; 2012b). By applying flexible offsetting schemes and sufficient multipliers, no net loss is considered achievable.

This consequentialist reasoning, however, is thought by some to be potentially self‐defeating. First, the claim that offsetting results in benefits to biodiversity and people is thought to legitimize exploitation as a benefit (Walker et al. 2009; Ives & Bekessy 2015), which some case studies confirm (Cowell 2000). Second, offsetting could lead to less government funding of traditional conservation (Hannis & Sullivan 2012). Third, if multipliers are too low, they risk signaling that biodiversity is expendable (Bull et al. 2017a). Rejecting these claims, proponents refer to the mitigation hierarchy, which may prevent it from being self‐defeating (BBOP 2012a; 2012b; Schoukens & Cliquet 2016). If correctly applied, the hierarchy allows compensatory offsetting only after the granting of a permit for exploitation. However, as noted by Apostolopoulou and Adams (2017), there is still a risk that the very existence of an offsetting option may lead to “an underuse of the earlier stages of the mitigation hierarchy.” Table 2 contains a summary of and comments on these various arguments and counterarguments to biodiversity offsetting.

**Table 2:** Summary of objections and counterarguments related to biodiversity offsets

Objections to offsetting	Arguments in favor of offsetting	Comments and potential agreement
Offsetting violates nature's intrinsic value: Dividing nature into tradeable units wrongly treats it as a means to human welfare, not as an end in itself.	Even if nature has intrinsic values, offsetting is sometimes needed to protect these values in practice.	The objection is countered consequentially rather than refuted. A common view to protect intrinsic, nonanthropocentric, nature values may still exist.
Losses of nature cannot be compensated for by human interventions: True fungibility does not exist for biodiversity or is very difficult to accomplish.	Full compensation is seldom claimed and not what offsetting aims for. Moreover, restrictions on trading are possible to implement.	Here, the counterargument refutes the objection. Opponents and proponents to offsetting may still agree though on restrictions and flexibility.
Too little is known to make adequate trades: It is wrong to offset when so little is known about nature and how to compensate over time.	If not always possible, offsetting is still functioning in many situations. By calculating with multipliers, safety margins can also be implemented.	Common views may exist in very complex and very simple cases. Disagreements more likely concern intermediate cases. Multipliers may still be agreed.
Offsetting impedes virtuous dispositions toward nature: If harm is not considered wrong per se, more damage and noncaring attitudes may result.	Being involved in offsetting activities may foster environmental awareness, including line with the mitigation hierarchy.	Opponents and proponents make different assumptions. Even if contextual attitudes can be measured with the aim to seek common views.
Offsetting has negative justice implications: The distribution of costs and benefits over space and time may create winners and losers.	Offsetting may increase overall utility, which opens for redistribution. Participation and multipliers enable sufficient compensation.	Opponents focus on practical outcome may differ with proponents theoretical ideas, but in many cases common ground could be identified.

Although our literature study revealed several views on offsetting, these were seldom explicitly linked to ethical concepts and paradigms, which obscures underlying values. The lack of ethical specificity may also prevent seeing nuances in arguments. In fact, ongoing discussions about conservation point to a panorama of positions. In response to a debate around the so‐called new conservation (Kareiva & Marvier 2012), 4 scholars started the Future of Conservation Project, aiming to “explore the views of conservationists on a range of issues” (Sandbrook et al. 2019). The project's work has shown that many conservationists actually do not exclusively belong to any one of the 4 main camps (traditional conservation, new conservation, critical social science, and market ecocentrism), each of which has allegedly polarized positions on conservation, but instead hold various combinations of views. Concerning offsetting, the traditional‐conservation orientation, which focuses on the protection of nature for its own sake, is most visible in the objection that offsetting violates nature's intrinsic value, but it figures in other arguments as well. The critical social‐science orientation, placing human well‐being at the forefront of conservation, is visible in the objection that offsetting may have unjust implications, but it also forms part of some of the other objections, including that offsetting impedes virtuous dispositions toward nature. The new‐conservation and market‐ecocentrism orientations, to the contrary, are both less critical of offsetting and regard market‐based tools as important, which does not necessarily imply lower conservation goals. In line with the conclusion of Sandbrook et al. (2019)—that many conservationists combine these views—some scholars argue that it is time to move beyond the debate about nature's intrinsic versus instrumental values. Tallis and Lubchenco (2014), for instance, defend a pluralistic conservation ethic that accepts all forms of value and welcomes all normative efforts to justify nature protection and restoration. In line with this, some points of potential agreement are described in Table 2.

In practice, offsetting schemes are often influenced by both deontological and consequential ethical considerations. Offsets may occur in combination with both no‐go areas and flexibilities. The former instrument is often justified, at least implicitly, on deontological grounds—there are limits to which entities should be included in offsetting. The latter instrument is applied on consequential, often utilitarian, grounds because it is believed to result in the best overall outcome, given that exploitation takes place. This again supports the calls from Sandbrook et al. (2019) to move beyond simplistic characterizations and from Tallis and Lubchenco (2014) to embrace a pluralistic conservation ethic.

In line with this ambition, we devised recommendations for policy makers and questions for practitioners linked to each of the 5 ethical objections to offsetting identified here (Table 3). For policy makers—often politicians or heads of public agencies—we formulated 10 recommendations on policy and legislation that we see as important to consider when opting for offsetting as a conservation instrument for promoting no net loss or net gains of biodiversity. Depending on the governance context, both soft and hard policy instruments may be needed, from legal provisions that limit the types of permissible trades (e.g., no go areas) to various guidelines issued by governments or public agencies. For the latter group—often consultants or local stakeholders—we formulated 5 questions, which mainly focus on process‐related issues, to reflect on when implementing offsetting in specific cases.

**Table 3:** Recommendations for policy makers and questions for practitioners regarding biodiversity offsetting

Objection	Recommendations for policy makers	Questions for practitioners
Offsetting violates nature's intrinsic value	The aims of biodiversity offsetting should be explicitly expressed in law and policy. Any specific situations when offsetting may not be used at all should be stated in law.	Are there case‐specific aspects that need to be particularly considered, such as unique biodiversity values that need to be fully respected?
Losses of nature cannot be compensated for by human interventions	The potential use of flexibilities (in type, space, and time) should be governed by policies. Any associated restrictions, for example, in terms of no‐go areas, should be stated in law.	How can offsetting be designed to prevent or limit negative impact on biodiversity and socioeconomic parameters?
Too little is known to make adequate trades	The use of multipliers, for precautionary purposes, should be promoted by policies. Limitations due to uncertainty and high complexity should first be legislated.	Can the case‐specific knowledge be improved, and can incremental steps and the use of multipliers reduce uncertainty?
Offsetting impedes virtuous dispositions toward nature	The value of biodiversity should be broadly promoted, including in educational policies. Impact on attitudes of potential habitat banking needs to be included in impact assessments of such systems.	How can learning among developers and the public be promoted in specific cases of offsetting, and how can attitudes over time be measured?
Offsetting has negative justice implications	The use of multipliers, for socioeconomic reasons, should be promoted by policies when relevant. Policies should promote public participation in decision‐making processes in cases of high societal relevance.	How can negative social implications be assessed, prevented, and managed, including through stakeholder engagement and use of multipliers?

We hope that the advice presented in Table 3 will help stimulate further and more thorough discussions of the ethics of biodiversity offsets. The design and implementation of offsetting policy does indeed reflect central values linked to biodiversity and human welfare. Respect for these values will increase as ethical clarity and analysis improve.
